# Temporal and regional incidence of carbapenemase-producing Enterobacterales, Switzerland, 2013 to 2018

**DOI:** 10.2807/1560-7917.ES.2021.26.15.1900760

**Published:** 2021-04-15

**Authors:** Alban Ramette, Michael Gasser, Patrice Nordmann, Reinhard Zbinden, Jacques Schrenzel, Damir Perisa, Andreas Kronenberg

**Affiliations:** 1 Swiss Centre for Antibiotic Resistance (ANRESIS), Institute for Infectious Diseases, University of Bern, Bern, Switzerland; 2 These authors contributed equally to the manuscript; 3 Molecular and Medical Microbiology, Department of Medicine, University Fribourg, Fribourg, Switzerland; 4 Institute for Medical Microbiology, University of Zurich, Zurich, Switzerland; 5 Laboratory of Bacteriology, Geneva University Hospitals, Geneva, Switzerland; 6 Federal Office of Public Health, Division of Communicable Diseases, Bern, Switzerland

**Keywords:** Carbapenemase-producing Enterobacterales, CPE, Switzerland, temporal and regional trends, genotypes, outbreak

## Abstract

**Introduction:**

In contrast to countries where carbapenemase-producing Enterobacterales (CPE) are endemic, only sporadic cases were reported in Switzerland until 2013. An aggravation of the epidemiological situation in neighbouring European countries indicated the need for a surveillance study in Switzerland.

**Aim:**

We aimed to describe CPE distributions in Switzerland and identify epidemiological factors associated with changes in incidence.

**Methods:**

Data on all human CPE isolates from 2013 to 2018 were collected by the Swiss Centre for Antibiotic Resistance (ANRESIS) and analysed for temporal and regional trends by Generalised Poisson regression. Isolates associated with infection or colonisation were included in a primary analysis; a secondary analysis included invasive isolates only. Statistical detection of regional clusters was performed with WHONET/SaTScan.

**Results:**

We analysed 731 CPE isolates, of which 325 (44.5%) were associated with screenings and 173 (23.7%) with infections. Yearly detection of CPE isolates increased considerably during the study period from 65 to 212. The most frequently isolated species were *Klebsiella pneumoniae* (54%) and *Escherichia coli* (28%). The most frequent genotypes were OXA-48 (43%), KPC (21%) and NDM (14%). In contrast to the French-speaking parts of Switzerland (West, Geneva) where OXA-48 were the predominant genotypes (around 60%), KPC was the most frequently detected genotype in the Italian-speaking region (63%). WHONET/SaTScan outbreak detection analysis identified seven clusters in five regions of Switzerland.

**Conclusions:**

In a first continuous surveillance of CPE in Switzerland, we found that the epidemiological situation aggravated nationwide and that regional patterns of CPE genotypes mirrored the situation in neighbouring European countries.

## Introduction

Because of their multidrug resistance, carbapenemase-producing Enterobacterales (CPE) cause increased mortality, morbidity and healthcare costs worldwide [1-3]. As carbapenemase genes are mostly plasmid-encoded and associated with various mobile genetic structures, they are transferred both vertically and horizontally, enabling rapid dissemination [4,5]. Based upon amino acid sequence similarities, carbapenemases are classified as members of either the Ambler class A including the *K. pneumoniae* carbapenemases (KPC), class B including Verona integron–encoded metallo-β-lactamase (VIM), Imipenemase (IMP) and New Delhi metallo-β-lactamase (NDM), or class D including several oxacillinases (OXAs) [6].

In the last decade, infections and colonisations with CPE have increased in the majority of European countries [7]. However, there are large differences regarding surveillance activities and reporting of different carbapenemase genotypes in different regions: KPC endemicity was reported for Greece and Italy, VIM has also been extensively reported in southern Europe, predominantly in Greece, interregional NDM spread has been reported in Poland, Romania and Denmark, and OXA-48 is endemic in Turkey and Malta and widespread in some western European countries (Belgium, France, Spain) [8,9].

In Switzerland, NDM carbapenemase producers were described for the first time in 2009 [10]. KPC [11] and OXA-48 [12] strains were identified one year later. All these reported cases were associated with single occurrences, without evidence of local spread, and were judged as likely introductions via patients from countries with endemic CPE. In 2013, Switzerland participated in the European Survey on Carbapenamase-Producing Enterobacteriaceae (EuSCAPE) project [13], which aimed to create a network of reference/expert laboratories able to provide information for monitoring the spread of CPE in Europe. At that time, agreed criteria for submitting CPE isolates to national expert laboratories had been adopted in Switzerland, but several weaknesses in the surveillance were identified such as the lack of a reference laboratory. The EuSCAPE group considered the epidemiological situation regarding CPE in Switzerland as moderate (stage 2b, ‘sporadic hospital outbreaks’), although an increasing trend was noticed. Since then, the epidemiological situation has become more critical in France and Italy, countries which border Switzerland [7]. 

In Switzerland, the number of local CPE outbreaks and imported cases has also increased (e.g. [14-16]) and based on these indications, the Swiss Federal Office of Public Health defined CPE as notifiable pathogens in 2016. However, no systematically collected epidemiological Swiss data have been published until now. The aims of this study were therefore (i) to describe CPE distributions and trends of different genera and genotypes in Switzerland from 2013 to 2018 on a national, regional and hospital level, including the characterisation of individual outbreak clusters, and (ii) to identify epidemiological factors associated with changes in case incidence. Because of major reorganisation of data collection at the national level in 2019, data after 2018 was not included in this analysis. 

## Methods

### Data acquisition

In 2013, the Swiss Antibiogram Committee (SAC) of the Swiss Society for Microbiology defined nine Swiss expert laboratories, accredited for characterising CPE on molecular level and connected to the Swiss Centre for Antibiotic Resistance ANRESIS. From 2013 to 2015, all Swiss primary laboratories from the eight regions of Switzerland (national population 8.5 million by the end of 2018) used in ANRESIS were asked to send all suspected human CPE isolates, irrespective of their origin (hospital or outpatient, invasive or colonising), to one of the expert laboratories for confirmation and molecular characterisation (data from individual regions are available on https://www.anresis.ch). For suspicion of CPE, primary laboratories used the guidelines and breakpoints of the given years from the European Committee on Antimicrobial Susceptibility Testing (EUCAST) or the Clinical and Laboratory Standards Institute (CLSI). As hospital outbreaks were rare so far in Switzerland, the Swiss National Center for Infection Control (Swissnoso) published hospital hygiene recommendations in 2017 that specified to perform screenings only for patients who had been hospitalised in foreign countries in the previous 12 months or for individuals with direct contact with CPE carriers [17]. For each isolate the persons in charge at the hospitals and primary laboratories collated data on specimen type, patient sex, age, place of residence, nationality and possible hospitalisation. The expert laboratories provided information on genotype based on PCR or sequencing. Data from non-carbapenemase-producing carbapenem-resistant Enterobacterales were excluded from further analyses. 

From 1 January 2016, reporting of all CPE isolates to the Swiss Federal Office of Public Health (SFOPH) became mandatory and SFOPH started collecting additional epidemiological data on stay abroad in the previous 12 months, prior medical interventions or contacts to other CPE carriers. Finally, the Swiss Centre for Antibiotic Resistance ANRESIS collated all data from 2013 to 2018. Deduplication was performed at the level of bacterial species, by keeping only the first date of occurrence of an isolate for a given year from the same patient. Because of possible horizontal transfer of CPE genes across bacterial species, a separate deduplication was performed on the CPE genotype level. ‘Infection only’ isolates were labelled as such according to their clinical classification.

### Statistical analyses

The number of CPE isolates was modelled via Generalised Poisson regression with year, regions, specimen types (stool, blood, respiratory tract, urine, wound, other) and sex as explanatory variables. Robust standard errors for the parameters were calculated according to Cameron and Trivedi [18], p values and 95% confidence intervals using the parameter estimates and their robust standard errors were calculated accordingly. Population size data was obtained from the Swiss Federal Statistical Office. All analyses and visualisations were done with the R software environment (version 3.3.2) [19].

### Cluster analyses

Cluster analyses were performed with WHONET 5.6 which embeds the SaTScan software. Simulated prospective space-time permutation scan statistics were calculated [20] with a baseline of 365 days. The maximum cluster length was set to 100 days. Recurrence intervals (i.e. the timespan in which an observed cluster can be observed once by chance) were calculated using 9,999 Monte Carlo simulations and considered significant if they were equal to or longer than 365 days. Single-case clusters containing different species were not considered. Isolates that were part of significant clusters were inspected at the level of raw data in detail. We then contacted infectiologists from the hospitals in which clusters were detected to confirm the identification of local outbreaks.

### Sensitivity analyses

Because the laboratories that sent CPE data in the period 2016 to 2018 (SFOPH data; 22 laboratories) did not perfectly match those that sent data to the expert network before (SAC data 2013–2015; 30 laboratories), a sensitivity analysis was performed that considered only the 18 laboratories that sent data during the whole study period. In addition, a sensitivity analysis was performed by considering infections only, i.e. by removing CPE isolates associated with CPE screenings.

### Ethical statement

This study was based on national surveillance data submitted to the Swiss Centre for Antibiotic Resistance ANRESIS. Because of the anonymous nature of the data, neither ethical approval nor written informed consent from patients were required.

## Results

After deduplication by species, a total of 731 single CPE isolates from 34 primary laboratories were recorded in Switzerland from 2013 to 2018 (Table 1). Among them, 325 (45%) and 173 (24%) isolates were associated with colonisation and infection, respectively, and for the remaining 233 this information was not available. A total of 454 (62%) isolates were from male patients and 666 (91%) were from patients older than 20 years. Of all characterised isolates, 286 (39%) were from stool, followed by urine (157; 21%), respiratory tract (40; 6%), and 19 (3%) from blood samples. In another deduplication which was performed by genotype only, 682 CPE isolates were counted.

**Table 1:** Characteristics of CPE isolates (deduplicated by species), Switzerland, 2013–2018 (n = 731)

	2013	2014	2015	2016	2017	2018	Total	Total (%)^a^
Clinical importance								
Colonisation	23	22	59	73	63	85	325	44.5
Infection	12	11	28	31	26	65	173	23.7
NA	30	49	29	38	25	62	233	31.9
Sex								
Female	20	29	36	54	51	74	264	36.1
Male	45	52	77	85	63	132	454	62.1
NA	0	1	3	3	0	6	13	1.8
Age group (years)								
0–4	6	1	4	7	5	6	29	4
5–19	3	1	4	2	4	8	22	3
20–64	29	42	59	68	50	89	337	46.1
≥ 65	27	37	42	64	55	104	329	45
NA	0	1	7	1	0	5	14	1.9
Specimen type								
Blood	3	2	3	3	3	5	19	2.6
Wound	1	4	7	14	6	11	43	5.9
Respiratory	4	2	10	12	4	8	40	5.5
Urine	10	26	26	29	24	42	157	21.5
Stool	22	25	44	73	51	71	286	39.1
NA	25	23	26	11	26	75	186	25.4

NA: not available.

^a^ Percentage from the total number of isolates (n = 731).

**Figure 1:**
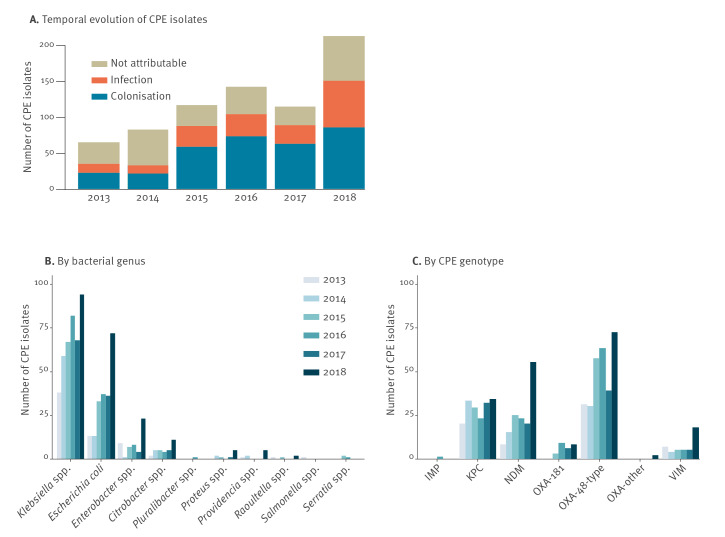
Evolution over time of CPE isolates related to colonisation and infection, Switzerland, 2013–2018 (n = 731)

From 2013 to 2018, the total number of CPE isolates increased more than threefold at the national level and went from 65 to 212 (+226%) yearly isolates (Figure 1A). While the total number seemed to have stabilised from 2015 to 2017 at around 120 CPE isolates yearly, the number increased drastically from 114 CPE isolates in 2017 to 212 in 2018 (+86%). This increase was to a considerable extent due to a larger number of CPE-associated infections in 2018 (increase from 26 to 65 isolates, +150%). In contrast, the number of isolates related to colonisation remained stable after 2014.

CPE: carbapenemase-producing Enterobacterales.

The category ‘OXA-other’ includes the few isolates with OXA-232 and OXA-244 genotype (Supplementary Table S2).

CPE were most frequently encountered in *Klebsiella* spp. (56% overall), *Escherichia coli* (27%) and *Enterobacter* spp. (8%) (Figure 1B, Table 2). Relative proportions of *E. coli* increased from 20% in 2013 to 34% in 2018, whereas *Klebsiella* spp. decreased from 59% to 44%. *Enterobacter* spp. decreased from 14% to 11% (Supplementary Table S1). An increase in CPE numbers was observed in all genera in 2018 compared with 2017: from 68 to 94 isolates (+38.2%) for *Klebsiella* spp., from 36 to 72 isolates (+100%) for *E. coli*, from four to 23 (+475%) for *Enterobacter* spp., but also for other, usually less frequently observed CPE genera such as *Citrobacter* spp. (+120% from five to 11 isolates) or *Proteus* spp. (+400%, from one to five isolates). The most frequent carbapenemase genotypes were OXA-48 (43%; Figure 1C, Table 2), KPC (25%) and NDM (21%). Only a few VIM-like, OXA-181 and IMP-like carbapenemases were reported (6%, 4% and < 1%, respectively; Figure 1C, Table 2). The number of isolates was higher in 2018 than in 2017 for all major genotypes (Figure 1C, Supplementary Table S2). In 2018, OXA-48 producers increased from 39 to 72 isolates (+85%), NDM-like producers from 20 to 55 isolates (+175%) and VIM increased from five to 18 isolates (+260%). The predominant genotype in *E. coli* was OXA-48 (61%), whereas most *Klebsiella* spp. were of the KPC (40%) and the OXA-48 (38%) genotypes, while 17% of *Klebsiella* spp. were NDM genotypes (Table 2).

**Table 2:** Total number of CPE isolates per genus and genotype, Switzerland, 2013–2018 (n = 682)

		*Klebsiella* spp.		*Escherichia coli*		*Enterobacter* spp.		*Citrobacter* spp.		*Proteus* spp.		*Providencia* spp.		Others		Total
IMP	n	1		0		0		0		0		0		0		1
	%	100	< 1	0		0		0		0		0		0		0
KPC	n	153		9		5		1		0		1		2		171
	%	90	40	5	5	3	9	< 1	3	0	0	< 1	14	1	17	25
NDM	n	66		41		21		9		3		5		1		146
	%	45	17	28	22	14	40	6	28	2	33	3	71	< 1	8	21
OXA-181	n	7		17		0		1		0		0		1		26
	%	27	2	65	9	0		4	3	0		0		4	8	4
OXA-48	n	144		113		15		13		0		1		6		292
	%	50	38	39	61	5	28	5	41	0		< 1	14	2	50	43
OXA-other	n	1		1		0		0		0		0		0		2
	%	50	< 1	50	< 1	0		0		0		0		0		0
VIM	n	11		5		12		8		6		0		2		44
	%	25	3	11	3	27	23	18	25	14	67	0		5	17	7
Total	n	383		186		53		32		9		7		12		682
	%	56		27		8		5		1		1		2		100

CPE: carbapenemase-producing Enterobacterales; IMP: Imipenemase; KPC: *Klebsiella pneumoniae* carbapenemase; NDM: New Delhi metallo-β-lactamase; OXA: Oxacillinase; VIM: Verona integron–encoded metallo-β-lactamase.

Left cell: Percentages relative to total count per row; right cell: Percentages relative to total count per column.

The category ‘others’ includes *Pluralibacter* spp., *Raoultella* spp., *Salmonella* spp., *Serratia* spp. and unknowns. The category ‘OXA-other’ includes the OXA-232 and OXA-244 genotypes. For a non-aggregated view of species and genotypes, see Supplementary Table S3.

**Figure 2:**
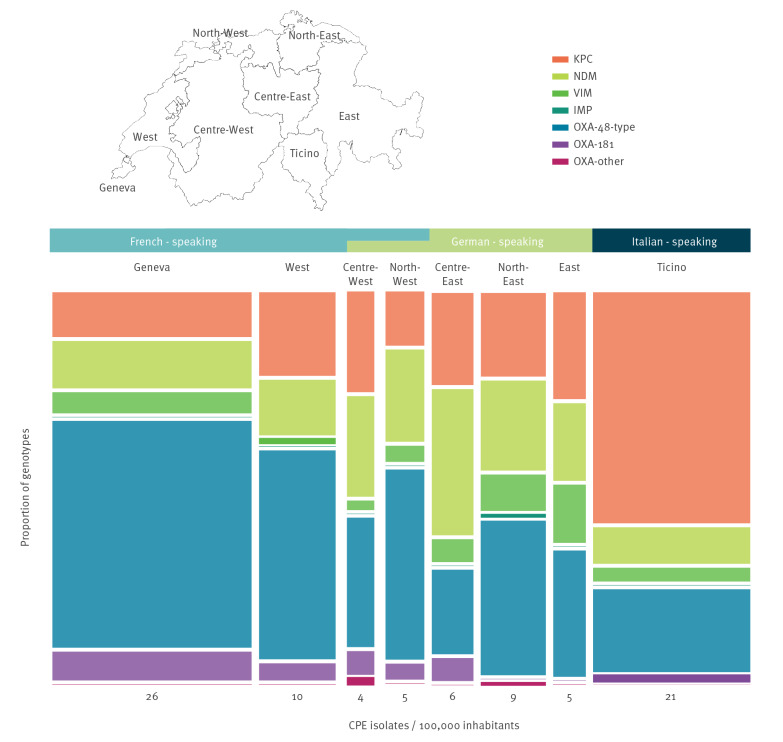
Geographical distribution of CPE genotypes per 100,000 inhabitants, Switzerland, 2013–2018 (n = 682)

CPE: carbapenemase-producing Enterobacterales; IMP: Imipenemase; KPC: *Klebsiella pneumoniae* carbapenemase; NDM: New Delhi metallo-β-lactamase; OXA: Oxacillinase; VIM: Verona integron–encoded metallo-β-lactamase.

The total number of CPE isolates increased over time in every Swiss region, except in the Centre-East and the Geneva region (Supplementary Table S4). The highest numbers of CPE isolates per 100,000 inhabitants were identified in the French-speaking region of Geneva and the Italian-speaking region of Ticino (Figure 2). In the latter area, the predominant genotype was KPC (63%), whereas in the French-speaking part including Geneva and the West, most isolates consisted of OXA-48 genotype (61% and 57%, respectively). In the predominantly German-speaking parts, OXA-48 was the most abundant genotype in all areas, except in the Centre-East region where NDM was the most abundant genotype (40%).

The mean for the whole country was eight isolates per 100,000 inhabitants over the whole study period.

### Multivariable analysis

**Figure 3:**
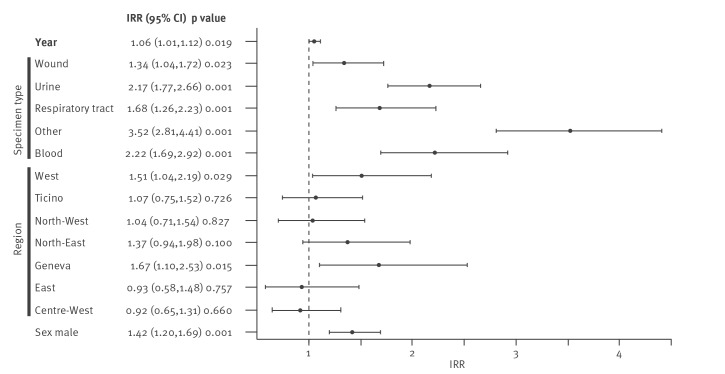
Factors affecting the number of CPE isolates in a multivariable Poisson regression Switzerland, 2013–2018 (n = 731)

CI: confidence interval; CPE: carbapenemase-producing Enterobacterales; IRR: incident rate ratio.

Multivariable analysis of regional and temporal CPE trends confirmed a temporal increase in total number. Compared with stool samples, all other types of specimen (wound, urine, respiratory tract, blood and others) were more likely to be associated with higher number of CPE isolates. The incidence was 1.42 higher in male than in female patients. The incidence rate ratio was 1.67 times higher in the Geneva region than in the Centre-East region where the number of CPE isolates was smallest (Figure 3).

Levels of reference were stool for the factor sample type, the Centre-East region (for the factor region) and female (for the factor sex).

### Cluster analysis

All 682 deduplicated genotypes from 2013 to 2018 were included in the simulated prospective cluster analysis by WHONET-SatScan. In five of eight regions of Switzerland, significant clusters were identified (i.e. the recurrence interval was > 365 days), resulting in a total of seven clusters (Table 3). In 2013 and 2014, only KPC clusters were detected, while thereafter, OXA-48 was the predominant genotype. All clusters had been identified by the responsible hospital infectiologists directly after their occurrence. Three of them were confirmed as locally detected outbreaks by genetic analyses, in the other three, no further investigations were performed because, among other reasons, they were considered as imported cases.

**Table 3:** Characterisation of CPE clusters detected by the automated WHONET-SatScan system, Switzerland, 2013–2018 (n = 682)

Signal period	22–28 Mar 2013	27 Sep–11 Oct 2013	2 May–12 Jun 2014	20 Mar–4 May 2015	1 Dec 2015	15–23 Feb 2018	25–28 Apr 2018
Genotype	KPC-2	KPC	KPC-2, KPC-3	OXA-48	OXA-48	OXA-48	VIM
Number observed	3	4	7	8	10	6	3
Number expected	0.5	0.55	1.32	1.94	2.44	0.91	0.13
Recurrence interval	559	588	2,113	667	16,540	720	655
Region	East	North-East	Centre-East	North-East	Geneva	West	North-East
Number of hospitals involved	1	2	1	1	1	1 or 2	1
Number of patients involved	3	4	7	6	5	3	3
Number of species involved	1	2	1	4	4	3	1
Confirmation status^a^	Cluster and outbreak confirmed	Cluster confirmed	Cluster confirmed, outbreak excluded	Cluster and outbreak confirmed	Cluster confirmed	Cluster confirmed	Cluster and outbreak confirmed

CPE: carbapenemase-producing Enterobacterales; KPC: *K. pneumoniae* carbapenemase; OXA: Oxacillinase; VIM: Verona integron–encoded metallo-β-lactamase.

^a^ Confirmation status indicates whether an accumulation of cases was detected in the hospital but no further investigation was performed (‘cluster confirmed’), whether a local outbreak was confirmed by genetic analyses (‘Cluster and outbreak confirmed’) or whether an accumulation of cases was detected but a local transmission was excluded by genetic analyses (‘Cluster confirmed, outbreak excluded’).

### Sensitivity analysis

The statistical analyses described above for the full dataset were repeated with the 166 isolates originating from infections only. Compositions of genotypes and species and the geographical distribution were largely identical in the two analyses (Supplementary Tables S5-S7, Supplementary Figure S1). No outbreak was detected in a cluster analysis by WHONET-SatScan considering only isolates from infections.

When considering the 18 laboratories that contributed CPE data in both the SAC database (2013–2015) and the SFOPH database (2016–2018), a total of 392 of 731 CPE species (53.6%), consisting of 378 (55.4%) of all 682 CPE genotypes, were retained and analysed. Results and trends reported from this analysis (Supplementary Figure S2) were largely congruent with the results described above (Figure 3). Noticeably, with the reduced dataset, the factor ‘year’ was not significant any more (p = 0.240).

## Discussion

The consolidation of CPE data from multiple, validated sources allowed us for the first time to study CPE rates over an extended period of time (2013–2018) in Switzerland. Hence, we examined not only the incidence and distribution of CPE on a national scale but also clustering at the regional and hospital level. The number of yearly detected CPE isolates has more than tripled during the study period. This increase could be confirmed, albeit to a lesser extent, in two subanalyses using infections only or considering laboratories participating throughout the whole study period, suggesting that the observed increase in isolates may not only be due to more frequent testing and/or reporting.

The increase in Switzerland mirrors the development in Europe, where a prevailing dissemination of CPE in healthcare systems has been described for 2010 to 2018 [7]. A closer look at the countries bordering Switzerland shows that the situation in Germany remained on an intermediate level (regional spread) between 2010 and 2018, whereas Italy entered in an endemic situation in 2013 and France reached a stage of inter-regional spread in 2014 [7,8]. The strong increase in Switzerland after 2017 may therefore be related to the epidemic situation in these neighbouring countries. In addition, the situation in Switzerland is probably affected by the deteriorating epidemiological situation in countries beyond its neighbourhood, from where a significant influx of CPE cases can be expected through (medical) tourism and migration. CPE isolates associated with *E. coli* doubled from 2017 to 2018 in Switzerland, but also isolates related to less common genera such as *Enterobacter* spp. and *Citrobacter* spp. increased substantially in the last year of our study. In particular the increased isolation of *E. coli* is worrisome and may indicate a dissemination of CPE in the community (not examined in our study), as already described in France [9]. Such a development would be of special concern as the spread of carbapenemase-producing bacteria in the community is more difficult to control than a spread in the hospital environment [5].

As reported from most European countries [13,21], *Klebsiella pneumoniae* was the most frequently observed CPE species in Switzerland. However, compared with other species, the increase of this typically nosocomial microorganism [21] was modest. Molecular data indicate that OXA-48, KPC and NDM types are the most prevalent genotypes in Switzerland. A clear majority of isolates belonged to the OXA-48 and OXA-48-like genotypes, which disseminated over all parts of Switzerland and most distinctly over the western region of Geneva. Possible explanations are the internationality of Geneva and the cross-border exchanges with Switzerland's western neighbour France where cases of OXA-48 and OXA-48-like genotypes have increased from regional to inter-regional spread since 2013 [8]. As OXA-48 and OXA-48-like genotypes do not exhibit high resistance levels to carbapenems, their detection is considered difficult and their real incidence could even be higher [5].

The stable occurrence of the KPC genotype goes along with the low increase in *K. pneumoniae* (as KPC was predominately found in *K. pneumoniae*). The incidence rates of KPC were highest in the Italian-speaking region of Ticino. This observation reflects well the situation in neighbouring Italy, where KPC-producing *K. pneumoniae* became endemic already in 2010 [8].

NDM increased considerably in 2018 in all regions of the country. This finding fits well with the worldwide prevalence of this genotype [22,23]. Equally remarkable was the recent sharp increase in VIM in Enterobacterales. However, the situation for VIM has to be monitored further in order to conclude whether it reaches an endemic stage as in southern Europe [8].

In the cluster analysis, the majority of detected outbreaks were related to the two most abundant genotypes OXA-48 and KPC. The shift from KPC- to OXA-48 clusters in 2014 and 2015 fits well with the increase in the OXA-48 isolates during the study period. Three of seven clusters detected by SatScan were confirmed as in-hospital outbreaks using genetic analyses conducted by local hospital infectiologists. Hence, we could demonstrate that a cluster analysis may facilitate the identification of real outbreaks. However, we suggest that such an approach needs to be done in combination with in-depth molecular typing technologies such as whole genome sequencing [24] in order to confirm cases that are genetically related. Other requirements for an accurate detection system for future outbreaks are the strict use of screening algorithms as published in 2017 by Swissnoso [17] and that all CPE isolates are sent to the same Swiss national reference laboratory (as done since 2019), which allows detailed and timely genetic analysis and thus the detection of potential inter-hospital transmission.

Limitations of our study include the fact that clinical and epidemiological data were not available throughout the whole study, e.g. not every isolate could be associated with infection or colonisation, and information about whether patients had stayed abroad was only available for some isolates after 2016. In addition, increased awareness (reflected in a change to mandatory reporting in 2016) could have artificially increased the number of detected CPE isolates during the study period. We believe, however, that our reported trends are robust, as the detected increase was confirmed by two subanalyses, which restricted the dataset to laboratories participating throughout the whole study period or to invasive isolates only.

The strengths of our study are that the analyses were based on data collected regularly over six consecutive years, covering all cultural and linguistic areas of Switzerland and that they included reports from the largest hospitals and laboratories nationally. With the inclusion of data from university, cantonal and regional hospitals, we can ensure that most hospitalised patients in Switzerland were represented.

## Conclusion

Our data describe a severalfold increase in incidence of all major CPE genotypes across all regions of Switzerland from 2013 to 2018. With OXA-48 as the predominant genotype in the western parts (bordering France) and KPC in the southern part (bordering Italy), the distribution within Switzerland mirrors the situation in western Europe. According to the trends in neighbouring European countries, we may also witness further spread of OXA-48 and NDM-producing *E. coli* and stabilisation or decrease of KPC producers. Owing to the current mandatory reporting scheme, a continuous surveillance of the situation in Switzerland is achieved, and a first important step has thus been taken to follow the progression of CPE incidence with high spatial and temporal resolution.

## Supplementary Data

150000 Supplement
