# Supplementary material for: Temporal and regional incidence of carbapenemase-producing Enterobacterales, Switzerland, 2013 to 2018
Source: Euro Surveill. 2021 Apr 15;26(15):1900760. doi: 10.2807/1560-7917.ES.2021.26.15.1900760 (PMC8167416; doi:10.2807/1560-7917.ES.2021.26.15.1900760)
Supplement: Supplement [file 19-00760_RAMETTE_supplement.pdf]

Supplementary material

This supplementary material is hosted by *Eurosurveillance* as supporting information alongside the article "Temporal and regional incidence of carbapenemase-producing Enterobacterales in Switzerland from 2013 to 2018", on behalf of the authors, who remain responsible for the accuracy and appropriateness of the content. The same standards for ethics, copyright, attributions and permissions as for the article apply. Supplements are not edited by *Eurosurveillance* and the journal is not responsible for the maintenance of any links or email addresses provided therein.

Table A1. Number of CPE isolates per species over time. Yearly marginal percentages are indicated in parentheses for each species. *Percentage to the total sum.

	2013	2014	2015	2016	2017	2018	Total
Citrobacter amalonaticus	0 (0)	0 (0)	0 (0)	0 (0)	1 (0.9)	0 (0)	1 (0.1)*
Citrobacter freundii	2 (3.1)	5 (6.1)	4 (3.4)	2 (1.4)	2 (1.8)	9 (4.2)	24 (3.3)
Citrobacter koseri	0 (0)	0 (0)	1 (0.9)	0 (0)	0 (0)	2 (0.9)	3 (0.4)
Citrobacter sedlakii	0 (0)	0 (0)	0 (0)	0 (0)	1 (0.9)	0 (0)	1 (0.1)
***Citrobacter* spp.**	0 (0)	0 (0)	0 (0)	2 (1.4)	1 (0.9)	0 (0)	3 (0.4)
Enterobacter aerogenes	0 (0)	0 (0)	1 (0.9)	1 (0.7)	1 (0.9)	1 (0.5)	4 (0.5)
Enterobacter cloacae	6 (9.2)	1 (1.2)	3 (2.6)	7 (4.9)	3 (2.6)	21 (9.9)	41 (5.6)
***Enterobacter cloacae* complex**	2 (3.1)	0 (0)	3 (2.6)	0 (0)	0 (0)	0 (0)	5 (0.7)
Enterobacter hormaechei	0 (0)	0 (0)	0 (0)	0 (0)	0 (0)	1 (0.5)	1 (0.1)
***Enterobacter* spp.**	1 (1.5)	0 (0)	0 (0)	0 (0)	0 (0)	0 (0)	1 (0.1)
Escherichia coli	13 (20)	13 (15.9)	33 (28.4)	37 (26.1)	36 (31.6)	72 (34)	204 (27.9)
Klebsiella oxytoca	0 (0)	0 (0)	2 (1.7)	3 (2.1)	4 (3.5)	6 (2.8)	15 (2.1)
Klebsiella pneumoniae	38 (58.5)	59 (72)	65 (56)	79 (55.6)	64 (56.1)	88 (41.5)	393 (53.8)
Pluralibacter gergoviae	0 (0)	0 (0)	0 (0)	1 (0.7)	0 (0)	0 (0)	1 (0.1)
Proteus mirabilis	0 (0)	2 (2.4)	1 (0.9)	0 (0)	1 (0.9)	4 (1.9)	8 (1.1)
Proteus vulgaris	0 (0)	0 (0)	0 (0)	0 (0)	0 (0)	1 (0.5)	1 (0.1)
Providencia rettgeri	1 (1.5)	0 (0)	0 (0)	0 (0)	0 (0)	3 (1.4)	4 (0.5)
Providencia stuartii	0 (0)	2 (2.4)	0 (0)	0 (0)	0 (0)	2 (0.9)	4 (0.5)
Raoultella ornithinolytica	1 (1.5)	0 (0)	1 (0.9)	0 (0)	0 (0)	1 (0.5)	3 (0.4)
***Raoultella* spp.**	0 (0)	0 (0)	0 (0)	0 (0)	0 (0)	1 (0.5)	1 (0.1)
Salmonella Kentucky	1 (1.5)	0 (0)	0 (0)	0 (0)	0 (0)	0 (0)	1 (0.1)
Serratia marcescens	0 (0)	0 (0)	2 (1.7)	1 (0.7)	0 (0)	0 (0)	3 (0.4)
**Unknown**	0 (0)	0 (0)	0 (0)	9 (6.3)	0 (0)	0 (0)	9 (1.2)
**Total**	65 (100)	82 (100)	116 (100)	142 (100)	114 (100)	212 (100)	731 (100)

Table A2. Number of CPE genotypes per year. Percentage contribution per year are provided in parentheses.

Genotype	2013	2014	2015	2016	2017	2018	Total
**IMP**	0 (0)	0 (0)	0 (0)	1 (0.8)	0 (0)	0 (0)	1 (0.1)
**KPC**	19 (28.8)	30 (36.6)	22 (18.5)	23 (18.5)	26 (25.5)	23 (12.2)	143 (21)
**KPC-2**	1 (1.5)	0 (0)	1 (0.8)	0 (0)	6 (5.9)	3 (1.6)	11 (1.6)
**KPC-3**	0 (0)	3 (3.7)	6 (5)	0 (0)	0 (0)	0 (0)	9 (1.3)
**KPC-3/19**	0 (0)	0 (0)	0 (0)	0 (0)	0 (0)	8 (4.2)	8 (1.2)
**NDM**	8 (12.1)	14 (17.1)	16 (13.4)	16 (12.9)	13 (12.7)	25 (13.2)	92 (13.5)
**NDM-1**	0 (0)	1 (1.2)	7 (5.9)	7 (5.6)	6 (5.9)	20 (10.6)	41 (6)
**NDM-19**	0 (0)	0 (0)	0 (0)	0 (0)	0 (0)	1 (0.5)	1 (0.1)
**NDM-4**	0 (0)	0 (0)	1 (0.8)	0 (0)	0 (0)	0 (0)	1 (0.1)
**NDM-5**	0 (0)	0 (0)	1 (0.8)	0 (0)	1 (1)	9 (4.8)	11 (1.6)
**OXA-181**	0 (0)	0 (0)	3 (2.5)	9 (7.3)	6 (5.9)	8 (4.2)	26 (3.8)
**OXA-232**	0 (0)	0 (0)	0 (0)	0 (0)	0 (0)	1 (0.5)	1 (0.1)
**OXA-244**	0 (0)	0 (0)	0 (0)	0 (0)	0 (0)	1 (0.5)	1 (0.1)
**OXA-48**	31 (47)	30 (36.6)	57 (47.9)	63 (50.8)	39 (38.2)	72 (38.1)	292 (42.8)
**VIM**	7 (10.6)	3 (3.7)	2 (1.7)	5 (4)	5 (4.9)	14 (7.4)	36 (5.3)
**VIM-1**	0 (0)	1 (1.2)	3 (2.5)	0 (0)	0 (0)	3 (1.6)	7 (1)
**VIM-4**	0 (0)	0 (0)	0 (0)	0 (0)	0 (0)	1 (0.5)	1 (0.1)
**Total**	66 (100)	82 (100)	119 (100)	124 (100)	102 (100)	189 (100)	682 (100)

Table A3. Total number of CPE isolates per species and genotypes for over six years. The marginal percentages per column and row, respectively are provided in parentheses.

Species	IMP	KPC	NDM	OXA-181	OXA-232	OXA-244	OXA-48-type	VIM	Total
Citrobacter amalonaticus	0 (0,0)	0 (0,0)	0 (0,0)	0 (0,0)	0 (0,0)	0 (0,0)	1 (0.3,100)	0 (0,0)	1 (0.1)
Citrobacter freundii	0 (0,0)	1 (0.6,4)	6 (4.1,24)	1 (3.8,4)	0 (0,0)	0 (0,0)	10 (3.4,40)	7 (15.9,28)	25 (3.7)
Citrobacter koseri	0 (0,0)	0 (0,0)	1 (0.7,50)	0 (0,0)	0 (0,0)	0 (0,0)	1 (0.3,50)	0 (0,0)	2 (0.3)
Citrobacter sedlakii	0 (0,0)	0 (0,0)	1 (0.7,100)	0 (0,0)	0 (0,0)	0 (0,0)	0 (0,0)	0 (0,0)	1 (0.1)
Citrobacter spp.	0 (0,0)	0 (0,0)	1 (0.7,33.3)	0 (0,0)	0 (0,0)	0 (0,0)	1 (0.3,33.3)	1 (2.3,33.3)	3 (0.4)
Enterobacter aerogenes	0 (0,0)	0 (0,0)	1 (0.7,33.3)	0 (0,0)	0 (0,0)	0 (0,0)	2 (0.7,66.7)	0 (0,0)	3 (0.4)
Enterobacter cloacae	0 (0,0)	5 (2.9,11.9)	17 (11.6,40.5)	0 (0,0)	0 (0,0)	0 (0,0)	10 (3.4,23.8)	10 (22.7,23.8)	42 (6.2)
Enterobacter cloacae complex	0 (0,0)	0 (0,0)	3 (2.1,50)	0 (0,0)	0 (0,0)	0 (0,0)	1 (0.3,16.7)	2 (4.5,33.3)	6 (0.9)
Enterobacter hormaechei	0 (0,0)	0 (0,0)	0 (0,0)	0 (0,0)	0 (0,0)	0 (0,0)	1 (0.3,100)	0 (0,0)	1 (0.1)
Enterobacter spp.	0 (0,0)	0 (0,0)	0 (0,0)	0 (0,0)	0 (0,0)	0 (0,0)	1 (0.3,100)	0 (0,0)	1 (0.1)
Escherichia coli	0 (0,0)	9 (5.3,4.8)	41 (28.1,22)	17 (65.4,9.1)	0 (0,0)	1 (100,0.5)	113 (38.7,60.8)	5 (11.4,2.7)	186 (27.3)
Klebsiella oxytoca	0 (0,0)	3 (1.8,23.1)	1 (0.7,7.7)	0 (0,0)	0 (0,0)	0 (0,0)	5 (1.7,38.5)	4 (9.1,30.8)	13 (1.9)
Klebsiella pneumoniae	1 (100,0.3)	150 (87.7,40.5)	65 (44.5,17.6)	7 (26.9,1.9)	1 (100,0.3)	0 (0,0)	139 (47.6,37.6)	7 (15.9,1.9)	370 (54.3)
Pluralibacter gergoviae	0 (0,0)	1 (0.6,100)	0 (0,0)	0 (0,0)	0 (0,0)	0 (0,0)	0 (0,0)	0 (0,0)	1 (0.1)
Proteus mirabilis	0 (0,0)	0 (0,0)	3 (2.1,37.5)	0 (0,0)	0 (0,0)	0 (0,0)	0 (0,0)	5 (11.4,62.5)	8 (1.2)
Proteus vulgaris	0 (0,0)	0 (0,0)	0 (0,0)	0 (0,0)	0 (0,0)	0 (0,0)	0 (0,0)	1 (2.3,100)	1 (0.1)
Providencia rettgeri	0 (0,0)	1 (0.6,25)	3 (2.1,75)	0 (0,0)	0 (0,0)	0 (0,0)	0 (0,0)	0 (0,0)	4 (0.6)
Providencia stuartii	0 (0,0)	0 (0,0)	2 (1.4,66.7)	0 (0,0)	0 (0,0)	0 (0,0)	1 (0.3,33.3)	0 (0,0)	3 (0.4)
Raoultella ornithinolytica	0 (0,0)	0 (0,0)	0 (0,0)	0 (0,0)	0 (0,0)	0 (0,0)	2 (0.7,66.7)	1 (2.3,33.3)	3 (0.4)
Raoultella spp.	0 (0,0)	0 (0,0)	1 (0.7,100)	0 (0,0)	0 (0,0)	0 (0,0)	0 (0,0)	0 (0,0)	1 (0.1)
Salmonella kentucky	0 (0,0)	0 (0,0)	0 (0,0)	0 (0,0)	0 (0,0)	0 (0,0)	1 (0.3,100)	0 (0,0)	1 (0.1)
Serratia marcescens	0 (0,0)	1 (0.6,33.3)	0 (0,0)	0 (0,0)	0 (0,0)	0 (0,0)	1 (0.3,33.3)	1 (2.3,33.3)	3 (0.4)
Unknown	0 (0,0)	0 (0,0)	0 (0,0)	1 (3.8,33.3)	0 (0,0)	0 (0,0)	2 (0.7,66.7)	0 (0,0)	3 (0.4)
Total	1 (0.1)	171 (25.1)	146 (21.4)	26 (3.8)	1 (0.1)	1 (0.1)	292 (42.8)	44 (6.45)	682 (100)

Table A4. Number of CPE isolates in different geographical regions of Switzerland 2013-2018. Column-wise percentages are provided in parentheses.

Region	2013	2014	2015	2016	2017	2018	Total
**Centre East**	1 (1.6)	11 (13.4)	3 (2.6)	15 (10.6)	5 (4.4)	11 (5.2)	46 (6.3)
**Centre West**	3 (4.7)	9 (11)	6 (5.2)	14 (9.9)	17 (14.9)	26 (12.3)	75 (10.3)
**East**	6 (9.4)	1 (1.2)	1 (0.9)	9 (6.3)	10 (8.8)	11 (5.2)	38 (5.2)
**Geneva**	18 (28.1)	14 (17.1)	37 (32.2)	28 (19.7)	13 (11.4)	22 (10.4)	132 (18.1)
**North East**	15 (23.4)	24 (29.3)	27 (23.5)	27 (19)	24 (21.1)	46 (21.7)	163 (22.4)
**North West**	6 (9.4)	7 (8.5)	14 (12.2)	6 (4.2)	11 (9.6)	31 (14.6)	75 (10.3)
**Ticino**	9 (14.1)	7 (8.5)	11 (9.6)	20 (14.1)	12 (10.5)	24 (11.3)	83 (11.4)
**West**	6 (9.4)	9 (11)	16 (13.9)	23 (16.2)	22 (19.3)	41 (19.3)	117 (16)
**Total**	64 (100)	82 (100)	115 (100)	142 (100)	114 (100)	212 (100)	729 (100)

Table A5. Number of CPE genotypes related to infections in the study.

Genotype	Infections (%)	All samples (%)
**IMP**	0 (0)	1 (<1)
**KPC**	43 (26)	171 (25)
**NDM**	38 (23)	146 (21)
**OXA-181**	5 (3)	26 (4)
**OXA-48-type**	66 (40)	292 (43)
**OXA-other**	2 (1)	2 (<1)
**VIM**	12 (7)	44 (6)
**Total**	166 (100)	682 (100)

Table A6. Number of CPE species related to infections in the study.

Species	Infections (%)	All samples (%)
Citrobacter amalonaticus	1 (0.6)	1 (0.1)
Citrobacter freundii	8 (4.6)	24 (3.3)
Citrobacter koseri	1 (0.6)	3 (0.4)
Citrobacter sedlakii	0 (0)	1 (0.1)
Citrobacter spp.	0 (0)	3 (0.4)
Enterobacter aerogenes	2 (1.2)	4 (0.5)
Enterobacter cloacae	15 (8.7)	41 (5.6)
Enterobacter cloacae Complex	3 (1.7)	5 (0.7)
Enterobacter hormaechei	0 (0)	1 (0.1)
Enterobacter spp.	0 (0)	1 (0.1)
Escherichia coli	43 (24.9)	204 (27.9)
Klebsiella oxytoca	3 (1.7)	15 (2.1)
Klebsiella pneumoniae	87 (50.3)	393 (53.8)
Pluralibacter gergoviae	0 (0)	1 (0.1)
Proteus mirabilis	5 (2.9)	8 (1.1)
Proteus vulgaris	1 (0.6)	1 (0.1)
Providencia rettgeri	1 (0.6)	4 (0.5)
Providencia stuartii	0 (0)	4 (0.5)
Raoultella ornithinolytica	0 (0)	3 (0.4)
Raoultella spp.	1 (0.6)	1 (0.1)
Salmonella Kentucky	0 (0)	1 (0.1)
Serratia marcescens	0 (0)	3 (0.4)
Unknown	2 (1.2)	9 (1.2)
**Total**	173 (100)	731 (100)

Table A7. Number of CPE cases per region related to infections in the study.

Region	Infections (%)	All samples (%)
Centre East	9 (5)	48 (7)
Centre West	20 (12)	69 (10)
East	9 (5)	37 (5)
Geneva	20 (12)	129 (19)
North East	40 (24)	166 (24)
North West	21 (13)	64 (9)
Ticino	24 (14)	74 (11)
West	23 (14)	93 (14)
**Total**	166 (100)	680 (100)

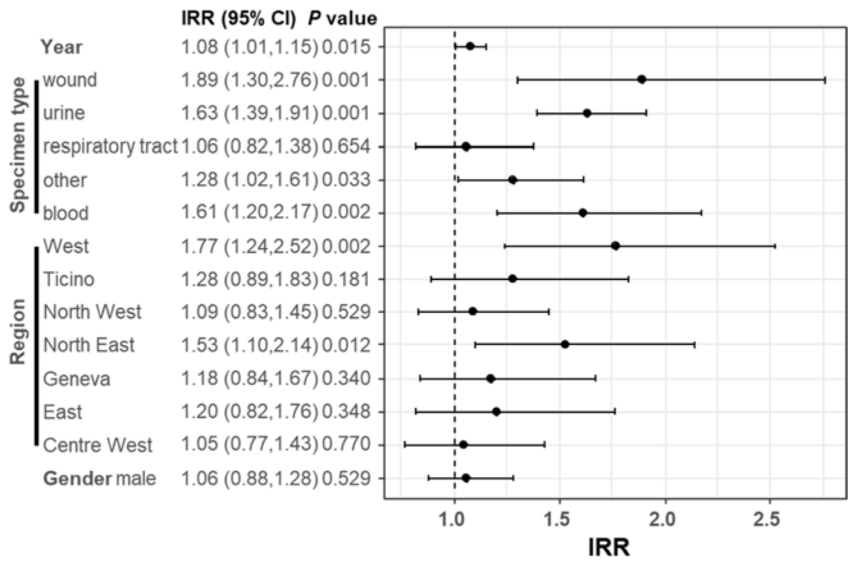

Figure A1. Factors affecting the number of CPE isolates for cases related to infections only. IRR: incident rate ratio; multivariable Poisson regression. The "infection only" samples were defined based on the classification given by "clinical importance" associated with the samples of origin, and not by the specimen type.

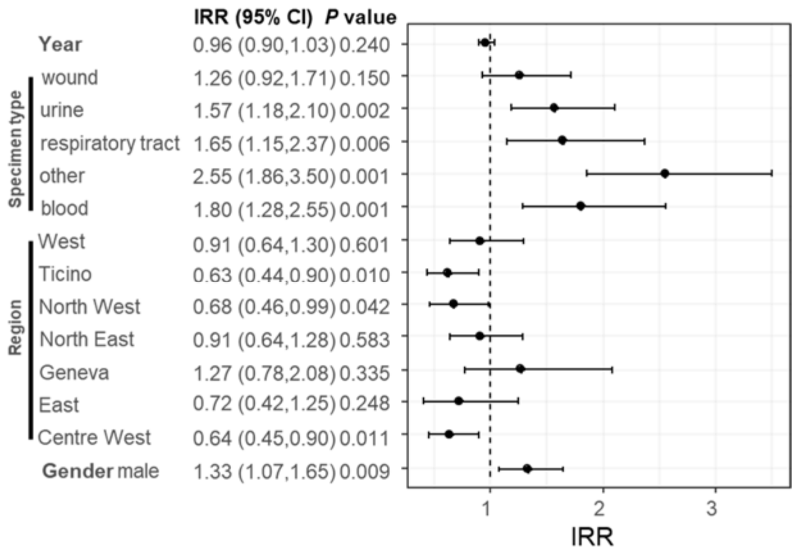

Figure A2. Factors affecting the number of CPE isolates including only the laboratories that sent data consistently from 2013 to 2018. IRR: incident rate ratio; multivariable Poisson regression
